# Integrating equity and social justice for indigenous peoples in undergraduate health professions education in Canada: a framework from a critical review of literature

**DOI:** 10.1186/s12939-021-01475-6

**Published:** 2021-05-21

**Authors:** Amlie Blanchet Garneau, Marilou Blisle, Patrick Lavoie, Catherine Laurent Sdillot

**Affiliations:** 1 Faculty of Nursing, Universit de Montral, 2375, chemin de la Cte-Sainte-Catherine, Montreal, Quebec H3T 1A8 Canada; 2 Faculty of education, Universit de Sherbrooke, 2500, boul. de lUniversit, Sherbrooke, Quebec J1K 2R1 Canada; 3 Department of anthropology, Cgep douard-Montpetit, 945, chemin de Chambly, Longueuil, Quebec J4H 3M6 Canada

**Keywords:** Equity, Social justice, Indigenous peoples, Undergraduate education, Health professions, Review, Curriculum

## Abstract

Understanding how to create structural change by actively counteracting racialized ways of interacting with Indigenous peoples at an individual and organizational level within health care systems and health professions education is essential for creating a more inclusive, equitable, and healthier society. In health professions education, the primary means of teaching about health inequities has been to frame them as stemming from culturally or ethnically based issues. While attention to culturally specific practices can be valuable to health and healing in some contexts, education that solely focuses on Indigenous cultures risks perpetuating cultural stereotypes and othering, rather than focusing on how Eurocentric systems continue to exert oppressive effects on Indigenous peoples. We present an organizational transformation framework grounded in equitable partnerships from a comprehensive critical review of the literature on the integration of equity and social justice in undergraduate health professions education with a focus on Indigenous health. We did a thematic analysis of the results and discussions presented in the 26 selected articles to identify promising practices and challenges associated with the integration of equity and social justice in undergraduate health professions education. The framework resulting from this analysis is composed of three interrelated components: 1) adopt critical pedagogical approaches that promote Indigenous epistemologies; 2) partner with Indigenous students, educators and communities; 3) engage educators in critical pedagogical approaches and health equity issues. This framework could guide the development of contextually tailored interventions that contribute to decolonizing health professions education.

## Introduction

To promote health equity for Indigenous peoples, the Truth and Reconciliation Commission of Canada (2015) recommends that all schools and faculties of medicine and nursing include Indigenous health issues in their curriculum. However, due to the deeply entrenched nature of colonization in current systems, educational and health institutions involved in health professions education are often poorly equipped to operationalize this recommendation. In fact, a recent literature review on the decolonization of post-secondary institutions in Canada shows that most focus predominantly on the inclusion of Indigenous people in existing structures [1]. This means that universities support the success of Indigenous students, staff and faculty, instead of transforming structures to include Indigenous knowledge systems and practices through a deep engagement with Indigenous peoples.

Some documented initiatives in Canada [2,5], the United States [6], and Australia [7, 8] focused on integrating Indigenous health content into the curriculum and made recommendations on program changes. However, authors emphasize the timeliness and sustainability issues of these initiatives as well as the lack of support from institutions. There is currently no study of the organizational processes needed to support the proposed transformations to integrate health equity for Indigenous peoples into programs, and thus ensure their acceptability and sustainability in academic institutions.

This paper reports on the results of a critical review of the literature aiming to critically appraise and synthetize the current state of knowledge relating to the integration of equity and social justice in the undergraduate health professions education with a focus on Indigenous health.

## Health equity and social justice in health professions education

The link between systemic racism towards Indigenous peoples and health disparities between Indigenous and non-Indigenous peoples in Canada is well documented [9]. Colonizing images of Indigenous peoplessuch as Indigenous mothers considered irresponsible and incompetent [10] or drunken Indians [11]prevail in Canadian society in the form of stereotypes. As a microcosm of society, health care facilities reflect the conversations that take place in the general public. Many health professionals, often unconsciously, view their interactions with Indigenous patients through the lens of these images and stereotypes of colonization. These dynamics become barriers to care and compromise the delivery of safe and equitable services [9, 12].

In health professions education, the primary means of teaching about health inequities has been to frame health and social inequities as stemming from culturally or ethnically based issues. While attention to culturally specific knowledge and practices can be valuable to health and healing in some contexts [13, 14], training that solely focuses on Indigenous cultures risks perpetuating cultural stereotypes and othering, rather than focusing on how Eurocentric systems continue to exert oppressive effects on Indigenous peoples. In nursing for example, the discourse remains grounded in individualism, which precludes collective commitment to injustices and their expression in race-based discrimination [15, 16]. The goal of addressing Indigenous health inequities through decolonizing care practices and structures of health systems has taken on a more prominent place in some health care and academic institutions, although many lack explicit strategies to foster health equity in policies, practices and procedures [13, 14, 17,21]. Decolonizing is to address the inequities brought about by colonization by recognizing First Nations peoples rights, autonomy, diversity, language, culture and our (Indigenous/non-Indigenous) shared histories, particularly by diminishing current power imbalances and the continuing impacts of structured privilege. [22].

Significant gaps have been identified in the integration of health equity and social justice into health professions education programs in Canada across all educational levels [23]. Often associated with the development of cultural competence, equity remains scarcely integrated into training programs and is often confused with equality. Health equity and social justice are core elements of the professional culture of health professionals [24, 25]. Health equity aims to address the social and health needs of populations by paying attention to those who are least served by the health system, which translates into a health status below the general population. Health equity differs from health equality, a guiding principle of health systems in most Western countries, which aims primarily to provide equal access to health care by assuming that people generally have the same opportunities and abilities to obtain quality care. The concept of health equity provides a useful analytical lens for examining systemic biases and addressing interpersonal and structural discrimination towards Indigenous peoples [26].

There are currently few tools to guide the integration of Indigenous health and health equity issues into training programs for future health professionals [3]. The few authors who have documented the integration of equity, social justice or content on systemic racism into health professions education programs emphasize the importance of incorporating these approaches and content in a longitudinal, cross-sectional and interdisciplinary manner from the outset of health professionals education [2, 4, 27]. Yet, literature reviews of Indigenous health training initiatives have shown that they most often consist of a single course, a collaborative workshop, a cultural immersion experience or a course module that rarely goes beyond one session [8, 28, 29]. Beavis, Hojjati et al. (2015) recommend the careful longitudinal and transversal integration of this content into programs in a context where Indigenous health curricula often appear to be opportunistically integrated in response to contingent requirements [8]. Both theoretical and practical social justice skills developed should be closely integrated with formal medical content in the compulsory curriculum and clinical modules [30], but also in optional courses for continuing development [31].

## A critical review of empirical studies

We conducted comprehensive, but not systematic, literature review of peer-reviewed empirical papers documenting the development, implementation or assessment of curricula based on anti-racist, anti-discrimination, post-colonial, equity or social justice approaches in the context of undergraduate health professions education. Keywords used for the searches are: (Anti-discriminatory OR antidiscriminat* OR Discriminat* OR Antiracist OR Racis* OR Prejudice OR stereotyp* OR oppression OR Social justice OR social equity OR social inequit* OR social responsibility OR societal injustice OR structural inequit* OR Intersectional* OR Structural competence OR Critical consciousness OR Postcolonial* OR Decoloni* OR Trauma-informed OR Anti-oppressive OR Ethnocentrism OR Vulnerab* OR Stigma* OR First Nation OR Indigenous OR Aboriginal OR Native OR Inuit OR Cultural safety) AND (Student* OR Curriculum OR Education OR Teaching method* OR Learning Method*) AND (Health sciences OR Social work OR nursing OR Pre-health OR Medical OR Occupational therapy OR Physiotherapy OR Pharmacy OR Psychology OR Dentistry OR Psychology). Searches limited to articles published in French or English from 2008 to 2019 and conducted in the CINAHL, ERIC and MEDLINE databases generated 647 results. After eliminating duplicates and reading abstracts, 228 articles were selected. From this preliminary list, articles were selected if their main focus was on the longitudinal and transversal integration of health equity, social justice, cultural safety, decolonizing, anti-racist or postcolonial approaches through a mandatory curriculum for undergraduate health professions students.

Research articles and literature reviews documenting one or more initiatives specifically related to Indigenous health were prioritized. Purely theoretical articles or editorials on the various approaches to health equity were excluded, as were articles documenting an exclusively intercultural approach to integrating cultural or transcultural competencies into a curriculum (due to their lack of critical perspective). Since we were looking for longitudinal and transversal integration of health equity related concepts in undergraduate education, articles were also excluded if the developed curriculum was solely aimed at graduate students (masters or doctoral levels), professors, or Indigenous students.

The 26 remaining articles (20 research articles and 6 literature reviews) were critically reviewed to highlight: the theoretical and pedagogical approaches underlying developed curricula; methods for building, implementing or assessing these initiatives; and the main conclusions and recommendations drawn from these experiences. We did a thematic analysis of the results and discussions presented in selected paper to identify promising practices and challenges associated with the integration of equity in initial training of health professionnals. From these themes, we could identify three interrelated components of the integration of equity and social justice: 1) adopt critical pedagogical approaches that promote Indigenous epistemologies; 2) partner with Indigenous students, educators and communities; 3) engage educators on critical pedagogical approaches and health equity issues. Health professions education programs mentioned in the selected articles included medicine, nursing, paramedical, community health, occupational therapy, pharmacy, physiotherapy, dentistry, psychology, and social work.

## An organizational transformation model grounded in equitable partnerships

**Fig. 1:**
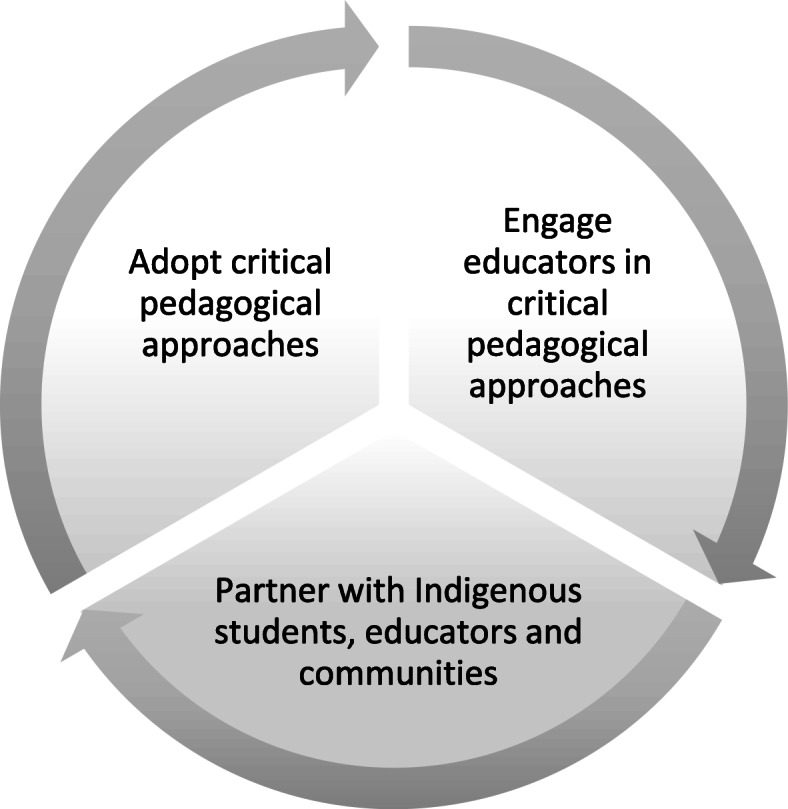
Integration of equity and social justice for Indigenous Peoples in health professions curriculum

The analysis highlighted a strong need for an organizational transformation process grounded in equitable partnerships to ensure the sustainable integration of equity and social justice for Indigenous peoples in health professions education. Based on the papers reviewed, changes in the training of future professionals are futile if they are not accompanied by profound institutional and organizational transformations, whether by implementing a curriculum on Indigenous health [32], social justice [31] or anti-racism [33]. A real willingness for change by institutions, evidenced by a formal policy or position statement, as well as the presence of senior leaders facilitating the maintenance of partnerships throughout the project are recognized as facilitating factors [4, 30, 31, 33, 34]. Thus, three interrelated and complementary conditions emerge from the reviewed literature for the sustainable integration of health equity for Indigenous peoples into training programs: 1) Adopt critical pedagogical approaches that promote Indigenous epistemologies; 2) partner with Indigenous students, educators and communities; 3) engage educators in critical pedagogical approaches and health equity issues (Fig.1.)

### Adopt critical pedagogical approaches that promote indigenous epistemologies

First, several researchers emphasized the importance of integrating critical perspectives that enable students to understand the societal and structural factors underlying health inequities [2, 27, 28, 35]. To bridge the health equity gap, it is essential to go beyond merely raising awareness of the negative experiences of Indigenous patients in the health system, and to aim to understand the broader racist discourses integrated into policies, structures and organizational cultures [36]. Some favour cultural safety approaches [4, 37], postcolonial approaches based on the decolonization of knowledge and practices [3, 6, 38], or anti-racist or intersectional approaches [2]. These critical perspectives focus on understanding colonial heritage and recognizing the power relationships that transcend health science culture and health care systems and practices [2]. For example, the cultural safety approach has been closely linked, for some, to the notion of cultural humility, a self-critical stance that involves recognizing ones biases and ethnocentrism to become aware of ones privileges [6], as well as accepting that the safety of care can only be judged by the people who receive it [2]. Integrating these approaches into programs and educational settings would allow students to develop a sense of responsibility and commitment to local communities [3, 6] and create safe learning, work and care environments for all [7, 39, 40]. These critical approaches are presented as complementary to or preferred over multicultural approaches [2, 6, 28, 41].

However, empirical studies revealed epistemological tensions, conceptual unclarity, and a problematic negotiation of space for Indigenous and critical content in the curriculum. It is difficult to define concepts that articulate the subtleties of the relationships between culture, society, geographic space and history [42]. According to Guerra and Kurtz [32], while the relevance of integrating cultural competence and cultural safety concepts into health training seems widely recognized, few programs developed to this end have been successfully implemented in Canada. These authors concluded that the significant variability in the skills associated with these concepts results in an equally wide diversity of programs developed with varying degrees of rigour. At the conceptual level, these inconsistencies and tensions have also been noted when comparing anti-racist educational programs or interventions [34]. The notions of race and social difference are sometimes addressed in critical terms of power and equity, sometimes as issues of cultural diversity and ethnicity [34]. It is thus recommended to clarify the concepts to be integrated [32] and to agree on desired student attitudes, values and skills [4, 43], as well as on the community needs assessment guidelines [43] and on the preferred program development framework [4, 32].

In a training context based on the Western health perspective, valuing and reconciling Indigenous knowledge and cultures with institutional care cultures is a major challenge [3, 37]. It proves complex to define a common vision of the best contents and approaches, considering the plurality of voices and perspectives of different stakeholders in working committees and collaborative groups [44]. It is therefore important to plan meetings to explore tensions between Indigenous and academic perspectives [37] and to recognize power inequalities [3]. The recognition and respect of the different approaches to organization and decision-making of both educational institutions and partner Indigenous communities form the basis of real collaboration [37].

These epistemological tensions are also reflected in negotiating the space given to Indigenous knowledge and critical (e.g. postcolonial, cultural safety, anti-racist) approaches in the training curricula of future professionals [3]. Decision-making committees, often consisting of people who share the Western paradigm, are primarily concerned with their disciplines accreditation standards [3]. For this reason, Beavis et al. (2015) consider it necessary for Canadian regulatory bodies and professional associations to commit to including postcolonialism in their standards of practice and accreditation and to subject it to formal assessments. In response to concerns about the place of a new cultural safety curriculum in dentistry, Bazen et al. (2008) succeeded in integrating the latter into existing units and the baccalaureate calendar. In the same vein, others have suggested that a relatively small number of targeted and structured teachings and learning can lead to significant changes in students knowledge, skills and attitudes [39].

The same issues were noted with regard to the development and implementation of curricula based on an anti-racist approaches [34]. Questioning the legitimacy of these approaches institutional power relations and the predominance of the culturalist approach (without emphasis on power and oppression dynamics) in institutional discourse are factors limiting change, while faculties commitment to standardizing anti-oppressive education, as well as changes to professional standards, are facilitating factors [34]. In-depth teaching of racism and awareness of privilege requires time and space, as well as further integration in all areas of medical training [35].

Valuing Indigenous knowledge and epistemologies includes ensuring that the content is taught by Indigenous teachers or staff [39], or that, at the very least, Indigenous voices are at the heart of teaching and learning approaches [28], that the acquisition of this knowledge is formally evaluated [2], and that elders are involved in both teaching [3] and mediating strategic sessions or collaborative groups, or even given an official role in programs [44]. Indeed, Mahara, Duncan [44] noted the positive impact of elder leadership in creating a safe community and environment during a strategic session. Several authors also suggest that, in addition to critical approaches, experiential learning principles should be incorporated into the developed program structure [2, 30, 32, 37, 43, 44]. When experiential learning is included, the administrative structure of programs should not only maintain relationships with communities but also monitor these projects to assess their impact on both communities and students. However, for Paul et al. (2006), rural immersion is not a necessary condition for transforming students consciousness and knowledge, since a large proportion of Indigenous people now live in urban settings.

### Partner with indigenous students, educators and communities

Stakeholder engagement is needed to secure partnerships between community and educational institutions beyond the funding period, which usually covers the initial phases of curriculum development and implementation [37]. Rowan et al. (2013) noted that most internal (between university departments and faculties) and external (between hospitals and communities) partnerships are very often informal (unless they include a student placement program). They also noted the lack of financial resources for integrating desired changes into curricula [5]. In addition, while collaborative curriculum development can sometimes occur without significant tensions between Indigenous and academic partners, tensions can arise in program administration (e.g. admission standards) or if other funding partners (e.g. government) impose new constraints and requirements [5].

To ensure the sustainability of the curriculum implementation and allow for constructive feedback, program development and implementation must be based on the principle of mutual partnership with Indigenous populations [2, 3, 5, 37, 44]. Conducted in accordance with the 4Rs: respect, relevance, reciprocity and responsibility and with a view to promoting the self-determination of Indigenous peoples [5], this type of partnership involves indigenizing processes by which content elements, learning objectives and pedagogical approaches are articulated and retained [6].

The development of programs to transform care for populations marginalized by health systems, whether Indigenous or not, should be a systematic process that meets both learner and community needs [43]. To this aim, some have used collaborative groups consisting of expert professors, Indigenous partners, community and academic stakeholders, and Indigenous health organizations [39, 40]. Others have relied on participatory approaches that promote community engagement [3] and mobilize interdisciplinary advisory groups through the iterative cycle of action research [6]. Beavis, Hojjati et al. (2015) recommend that communities be involved in the search for wise practices for teaching the developed content. The notion of wise practices, referring to locally appropriate actions, tools, principles or decisions that contribute significantly to the development of sustainable and equitable conditions [45], is here opposed to best practices that are considered reductionist given the heterogeneity of contexts where they are developed and [2]. Ambrose et al. (2014) value the development of the student-driven social justice curriculum, based on Paulo Freires principles of andragogy and transformative learning. Modelled by students, the curriculum can be evaluated and adjusted by each cohort at low cost [46]. However, the authors point out that the success of these curricula depends largely on the students who participate voluntarily, as well as on faculty collaboration and the support of the educational institution [46].

Thus, the engagement and collaboration of Indigenous faculty and students in curriculum development, facilitated by the institution, is critical to the success of initiatives [6]. This process of building curricula based on mutual partnership can take many forms, whether as one-off or one-time events such as off-campus strategic sessions [44], collaborative workshops [3] or symposia [42], or a collaborative approach involving a working group and spanning several months/years (e.g. Virdun et al. 2013). At the term of this process, it is important to recognize the co-constructed and co-authored nature of the produced model or framework [37], as well as to lay foundations to ensure the sustainability of this partnership and iterative cycle [3, 5, 44]. Conducted in a spirit of collaboration and reciprocity, these initiatives allow the emergence of a whole network of social relationships that can generate other positive projects for the future of communities [37]. In this respect, there is a strong need for clear recruitment and retention policies for Indigenous faculty and students [33, 37, 42].

### Engage educators in critical pedagogical approaches and health equity issues

Many educators are reluctant to address issues of race and racism, and actively avoid doing so [47, 48]. This discomfort is often attributed to a lack of knowledge or appropriate training [35]. Thus, in any redefinition of Indigenous health programs, teacher training should be prioritized to increase their engagement in the success and sustainability of these programs [3, 4, 6, 7, 24, 35]. This includes the use of conferences, strategic sessions and focus groups [4]. Often, when professional development activities in Indigenous health are optional for faculty, participation rates remain low [3]. Teacher training and mobilization vary greatly depending on personal knowledge, background, identity and access to appropriate support [3, 34]. Some non-native professionals and teachers may also feel fear and a sense of imposture about the need to teach Indigenous content [7].

It is therefore recommended that safe spaces (such as monthly discussion groups) be created where teachers can learn from each other to increase their sense of competence, and where Indigenous voices can be heard [7, 35]. Building on the knowledge and experiences of professors is a key to the successful implementation of Indigenous health curricula [39], as is the collaboration of leader-professors who are already successfully integrating this content into their courses [4, 31].

Professors must also be able to think critically about their pedagogical practices, the ethnocentrism of the biomedical model, and their commitment to communities in order to encourage students to do the same [2, 30, 43]. Studies have noted that students perceptions of the value of Indigenous health education vary widely, as do their emotional reactions to teaching content and approaches [28]. Diffey and Mignone (2017) noted that various factors can negatively influence students reception of anti-racist teachings, such as cultural isolation or racial tensions in a region, unequal power relations between students in the classroom, and learners identities. A desire to learn about the subject, a favourable classroom climate, clear discussion rules, students involvement in course planning, and reflective writing are, on the contrary, facilitating factors [34]. Limits in students receptiveness, ambivalent emotions, and feelings of discomfort are seen as opportunities to engage in real dialogue [44] and to generate profoundly transformative learning, although further research is needed to measure the impacts of a discomfort pedagogy [28].

## Issues and future direction for a sustainable integration of equity in health professions education

Implementation of the three interrelated components could be a first step in strengthening the capacity of educational institutions to train health professionals who can, through concrete actions, fight against individual and systemic discrimination and health inequities that are particularly targeted at Indigenous people, families and communities. However, an important challenge lies in the development of standards for curriculum evaluation and outcomes. Rowan et al. (2013) note that very few valid tools and indicators have been developed to measure student and faculty learning and attitudinal changes due to curriculum changes. Most often, impact assessment is based on students subjective perceptions of their integration of concepts and approaches [4], but fails to measure patient outcomes [8]. It is assumed that improving students skills, knowledge and attitudes will generally benefit the health of Indigenous populations [8]. Several researchers note the difficulties of measuring the practical impact of theoretical education on the health of vulnerable and racialized populations [31, 41, 46], and more specifically on Indigenous peoples health and equitable access to care [3, 32].

### Review limitations

This critical review of the literature was comprenhensive but not systematic and included peer-reviewed articles documenting the development, implementation or assessment of curricula based on anti-racist, anti-discrimination, post-colonial, equity or social justice approaches in the context of undergraduate health professions education. Consequently, effects of the pedagogical approaches such as the reduction of health inequities for Indigenous peoples, were not assessed in the selected papers. This should be taken into consideration while assessing and using the framework we propose as a result of this critical review. Further research is then needed to assess the impact of implementing this framework on future health professionnals clinical practice and on health inequities. For example, longitudinal studies that complement existing comparative and experimental studies would assess the long-term impacts of this learning and thus refine curricula [28, 39, 41, 43]. Jacklin et al. (2014) suggest until they are able to conduct these studies that Indigenous partners are mobilized to periodically re-evaluate developed curricula post-implementation. These researchers are conducting a preliminary assessment of the success of the program developed at the Northern Ontario School of Medicine based on the satisfaction of Indigenous partners and the strength and sustainability of relationships established with communities [3]. The same issues of lack of rigour and clear assessment standards for learning and its long-term impacts were found with regard to the integration of anti-racist content [34, 43].

## Conclusion

The results of this critical review have the potential to strengthen the ability of schools and faculties of nursing and other health fields such as medicine, occupational therapy and physiotherapy to redefine their training programs from an equity perspective for Indigenous peoples. Colonialism, which is still present in social structures and is at the root of systemic discrimination and inequities experienced by Indigenous peoples, is not unique to Canada, and its impact on health and the well-being of indigenous peoples is manifested worldwide [26]. The results of this analysis may therefore be international in scope by inspiring educational institutions training future health professionals elsewhere in the world. The knowledge developed in connection with processes to be implemented in health professions training is also useful for integrating issues related to racialized populations into programs since they are subject to the daily setbacks of colonization and the domination of knowledge and practices developed from a Western perspective [2].

## Data Availability

The data that support the findings of this study are available from Amlie Blanchet Garneau but restrictions apply to the availability of these data, which were used under license for the current study, and so are not publicly available. Data are however available from the authors upon reasonable request and with permission of Amlie Blanchet Garneau.
